# Travelers’ health problems and behavior: prospective study with post-travel follow-up

**DOI:** 10.1186/s12879-016-1682-0

**Published:** 2016-07-13

**Authors:** Katri Vilkman, Sari H. Pakkanen, Tinja Lääveri, Heli Siikamäki, Anu Kantele

**Affiliations:** Department of Bacteriology and Immunology, University of Helsinki, Haartmaninkatu 3, (P.O. Box 21), 00014 Helsinki, Finland; Inflammation Center, Clinic of Infectious Diseases, Helsinki University Hospital and University of Helsinki, Aurora Hospital, Nordenskiöldinkatu 20, (P.O. Box 348), Helsinki, Finland; Institute of Clinical Medicine, University of Helsinki, Helsinki, Finland; Aava Travel Clinic, Medical Centre Aava, Annankatu 32, 00100 Helsinki, Finland; Unit of Infectious Diseases, Solna, Karolinska Institutet, SE-171 76 Stockholm, Sweden

**Keywords:** Travel, Travelers’ health, Travelers’ behavior, Travelers’ diarrhea, Malaria, Antimalarials, Risk factors, Vaccinations, Antimicrobials

## Abstract

**Background:**

The annual number of international tourist arrivals has recently exceeded one billion, yet surprisingly few studies have characterized travelers’ behavior, illness, and risk factors in a prospective setting. Particularly scarce are surveys of data spanning travel, return, and follow-up of the same cohort.

This study examines behavior and illness among travelers while abroad, after return home, and at follow-up. Patterns of behavior connected to type of travel and illness are characterized so as to identify risk factors and provide background data for pre-travel advice.

**Methods:**

Volunteers to this prospective cohort study were recruited at visits to a travel clinic prior to departure. Data on the subjects’ health and behavior were collected by questionnaires before and after journeys and over a three-week follow-up. In addition, the subjects were asked to fill in health diaries while traveling.

**Results:**

The final study population consisted of 460 subjects, 79 % of whom reported illness during travel or on arrival: 69 % had travelers’ diarrhea (TD), 17 % skin problems, 17 % fever, 12 % vomiting, 8 % respiratory tract infection, 4 % urinary tract infection, 2 % ear infection, 4 % gastrointestinal complaints other than TD or vomiting, and 4 % other symptoms. Of all subjects, 10 % consulted a doctor and 0.7 % were hospitalized; 18 % took antimicrobials, with TD as the most common indication (64 %). Ongoing symptoms were reported by 25 % of all travelers upon return home.

During the three-week follow-up (return rate 51 %), 32 % of respondents developed new-onset symptoms, 20 % visited a doctor and 1.7 % were hospitalized.

Factors predisposing to health problems were identified by multivariable analysis: certain regions (Southern Asia, South-Eastern Asia, and Eastern Africa), female gender, young age, and long travel duration.

**Conclusions:**

Despite proper preventive measures like vaccinations, malaria prophylaxis, and travel advice, the majority of our subjects fell ill during or after travel. As the symptoms mostly remained mild, health care services were seldom needed. Typical traveler profiles were identified, thereby providing a tool for pre-travel advice. The finding that one third reported new-onset illness during follow-up attests to the importance of advising clients on potential post-travel health problems already during pre-travel visits.

**Electronic supplementary material:**

The online version of this article (doi:10.1186/s12879-016-1682-0) contains supplementary material, which is available to authorized users.

## Background

According to travel records for 2015, the number of international tourist arrivals exceeded one billion, and half a billion people headed to emerging economies [1]. Less than half of the visitors to poor regions have been shown to seek pre-travel health advice, as exemplified by only 15 % of Canadians visiting hepatitis A endemic countries [2] and 31 % of Australasians traveling to Asia, Africa, or South America [3]. This is surprising, considering the high morbidity rates (64–70 %) reported for visitors to developing regions [4, 5].

Investigations addressing travelers’ health have generally been retrospective and/or conducted among those seeking medical care after their journeys [6–21]. Not many prospective studies focus on the spectrum of travelers’ diseases [4, 5, 22–27]. Among these we found only one that looks at the same cohort during and after travel and also includes a post-travel follow-up [4]. The principal findings of the prospective studies are mildness of symptoms [4, 22] and small proportion of those falling ill who seek medical care: 2–33 % of the total study population visit a physician and 0.1–4 % are hospitalized [4, 5, 22, 24, 25, 27] during travel. After returning home, 9–20 % of all travelers have been reported to see a doctor [4, 5, 22, 25] and 0.0–1.0 % to be hospitalized [4, 22, 27]. Even though those with the most severe symptoms probably seek medical care, it should be noted that they represent only the tip of the iceberg among travelers falling ill. To get a comprehensive view of travel-associated health problems, prospective study designs should be employed for collecting data on illness while abroad, after return, and at follow-up.

To examine the health problems of travelers overseas, we recently conducted a nation-wide study of a large Finnish database provided by an assistance organization [21]. While these data presumably cover cases with the most severe symptoms, we sought to complete them with a prospective study that would comprise even mild illness. By including a post-travel follow-up, we extended the research to symptoms not developing until after return.

## Methods

### Volunteers and study design

**Fig. 1:**
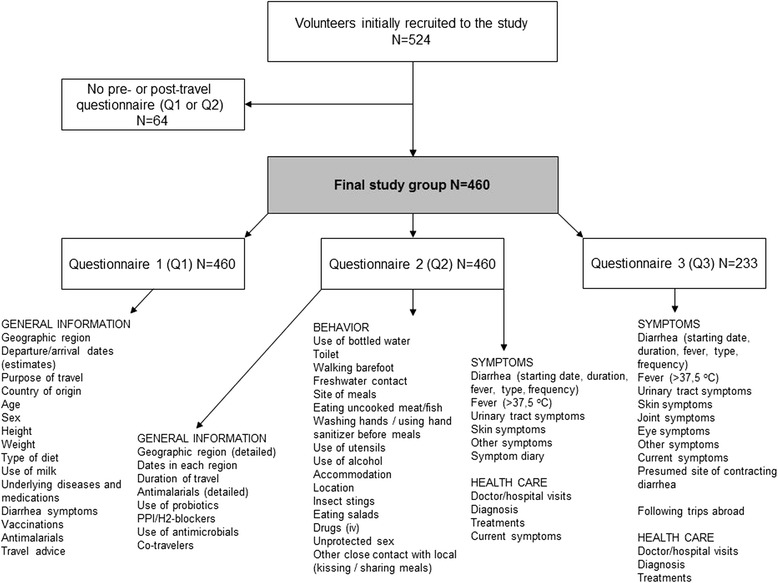
Study protocol, population, and information collected by questionnaires of a total of 524 travelers recruited at the pre-travel consultation

Volunteers to this prospective study cohort were enrolled at the Travel Clinic of Aava Medical Centre among travelers planning a journey outside the Nordic countries for a minimum of four days and a maximum of six months (Fig. 1). The only exclusion criterion was non-compliance in returning questionnaires. The subjects were recruited among consecutive clients at pre-travel appointments between December 2008 and February 2010. At the initial visit, they filled out a pre-travel questionnaire (Q1), on return home a post-travel questionnaire (Q2), and about three weeks later a follow-up questionnaire (Q3). Those who failed to return both Q1 and Q2 were excluded. In addition to the questionnaires, the subjects were asked to complete a structured diary on a voluntary basis. All volunteers were given pre-travel advice by a health care professional.

We have earlier reported risk factors for acquiring resistant intestinal microbes in the same population [28], and, data on diarrheal pathogens, first in our methodological investigation [29] and, recently, in an etiological study [30].

### Questionnaires

The questionnaires consisted of 134 multiple-choice or open-ended questions: Q1 comprised 47, Q2 60, and Q3 27 questions. These questionnaires were modified from a set of survey questions routinely used for more than 10 years for ill travelers admitted to the Clinic of Infectious Diseases at HUCH. All items covered are listed in Fig. 1. The structured diary contained more specific questions on duration and severity of symptoms, and use of antibiotics.

### Definitions

Subjects were classified as ill if they reported symptoms implying health problems which could be travel-related. Diarrhea was defined according to WHO criteria, *i.e.* passage of 3 or more loose or liquid stools per day, or more frequently than is normal for the individual (World Health Organization [31]). At follow-up, symptoms which had set in more than two days after a journey and could be travel-related were categorized as newly onset.

### Destinations

The countries visited were grouped into nine geographic regions (UN categorization, modified [32]): Southern Asia, South-Eastern Asia, Eastern Asia and Central Asia, Southern Africa, Eastern Africa, Western Africa and Middle Africa, Northern Africa and Western Asia, Latin America and the Caribbean, and Europe and Northern America (Table 1). The destination with the greatest health risk was considered primary for subjects traveling to several places. Here we used a rating based on the risk map drawn up by International SOS (Additional file 1: Figure S1 HealthMap 2010) which takes into account a range of factors: the standard of local medical and dental care, access to prescription drugs, the possible prevalence of serious infectious diseases, and known cultural, linguistic and administrative barriers.

**Table 1:** Demographics, travel information, and destinations of 460 travelers recruited at the travel clinic during their pre-travel visit, all staying outside the Nordic countries for more than four days and less than 6 months. The data are given as numbers of cases and their proportions

	Number	(%^a^)
Sex (missing data = 0)		
Male	173	(38)
Female	287	(62)
Age, median (IQR) (missing data = 0)	35 (27.0–54.0)	
0–17, children	35	(8)
18–35, young adults	196	(43)
36–55, middle aged	120	(26)
56–, older travelers	109	(24)
Underlying diseases (missing data = 0)	192	(42)
Asthma, atopy and/or allergy	56	(12)
Hypertension	51	(11)
Psychiatric disorder or medication	25	(5)
Cardiovascular disease	14	(3)
GI disorder ^b^	13	(3)
Neurological disorder	12	(3)
DM	9	(2)
Rheumatic disease	6	(1)
Other disease and conditions	78	(17)
Contraceptives or postmenopausal hormone replacement therapy	53	(12)
Duration of travel, median in days (IQR) (missing data = 2)	16 (12.8–27.3)	
1 week or less (missing data = 0)	27	(6)
More than a week and less than a month	334	(73)
1–5 months	99	(22)
Purpose of travel (missing data = 1)		
Vacation	383	(83)
Business	38	(8)
Other/multiple ^c^	38	(8)
Geographic region (missing data = 0) ^d^		
South-Eastern Asia	107	(23)
Eastern Africa	96	(21)
Western Africa, Middle Africa	86	(19)
Southern Asia	68	(15)
Latin America and the Caribbean	41	(9)
Southern Africa	26	(6)
Europe, Northern America	15	(3)
Northern Africa, Western Asia	12	(3)
Eastern Asia, Central Asia	9	(2)

^a^ Proportion of positive among those from whom information was available

^b^ Crohn's disease, irritable bowel syndrome, primary biliary cirrhosis, diverticulosis, microscopic colitis, celiac disease

^c^ 14 (3 %) travelers reported two or more purposes of travel

^d^ Given according to primary destination. Categorized into three subgroups: Asia (Western Asia, South-Eastern Asia, Southern Asia, Eastern Asia, and Central Asia), Africa (Eastern Africa, Western Africa, Middle Africa, Southern Africa, and Northern Africa), and the others (Latin America and the Caribbean, Europe, North America)

### Statistics

Univariable, bivariable and multivariable models were used. The p-value of Pearson Chi-square tests and Fisher’s exact test < 0.05 was considered statistically significant. If bivariable p-value was less than 0.10, the factor was chosen to the multivariable logistic regression model, and its adjusted odds ratios and 95 % confidence intervals were calculated. Missing data were assumed to be missing at random (MAR) and missing values were imputed using multiple imputations in SPSS. The statistical analyzes were carried out with SPSS Statistics (version 22.0.0.2, IBM Corp., USA).

## Results

### Cohort population and prophylactic measures

Of the 524 initially recruited travelers, 88 % completed both pre- and post-travel questionnaires. The final study population thus consisted of 460 volunteers (Fig. 1, Table 1). A total of 233 (51 %) returned follow-up questionnaires and 295 (64 %) filled in diaries. An underlying disease or condition was recorded for 192 (42 %)(Table 1), more frequently among the oldest subjects (56– years, 73 %; *p* < 0.001), and females (45 %; *p* = 0.046) than in other age groups (0–55 years, 23–38 %) or among males (36 %). Data on vaccinations are presented in Table 2 and on antimalarials in Table 3.

**Table 2:** Numbers and proportions of travelers (*n* = 460) vaccinated before the travel clinic appointment or at consultation. The data are given as numbers and percentages of those vaccinated or considered protected according to Finnish recommendations

	Protected before consultation, *N* (%^a^)	Vaccinated at consultation, *N* (%^a^)	Protected according to recommendations given for the region visited, *N* (%^a^)
Tetanus (missing data = 4)	353 (77)	96 (21)	449 (98)
MMR (missing data = 43)	393 (94)	21 (5)	414 (99)
Hepatitis A (missing data = 3)	325 (71)	130 (28)	440 (100)
Hepatitis B (missing data = 4)	178 (39)	124 (27)	72 (73)
Yellow Fever (missing data = 2)	112 (24)	153 (33)	182 (96)
Polio (missing data = 4) ^b^	140 (31)	84 (18)	104 (63)
Typhoid fever (missing data = 3)	38 (8)	63 (14)	47 (65)
oral	16 (4)	59 (13)	
injected	22 (5)	4 (1)	
Japanese encephalitis (missing data = 3) ^c^	15 (3)	30 (7)	18 (53)
Other (missing data = 3) ^d^	14 (3)	58 (13)	

^a^Proportion of positive among those from whom information was available

^b^Part of the volunteers may have chosen to skip polio vaccination during appointment at the travel clinic and, instead, get it from their health center where they are vaccinated free of charge since polio is included in the Finnish national immunization program

^c^Despite advice many declined prophylaxis because of high price

^d^Includes cholera, meningococcal, and rabies vaccinations

**Table 3:** Medications taken by 460 Finnish travelers during the trip. The medications are categorized according to indication. The data are presented as numbers and percentages of travelers

	Number	(%^a^)
Prophylactic antimalarial use among those recommended (missing data = 6)	289	(98)
Atovaquone and proguanil	135	(30)
Mefloquine	70	(15)
Doxycycline	55	(12)
Chloroquine	25	(6)
Other / not known	2	(0)
Changed drug ^b^	2	(0)
Failure in use ^c^	7	(2)
Antimicrobials (other than doxicycline) (missing data = 0) ^d^	81	(18)
Fluoroquinolone	53	(12)
Macrolide	9	(2)
Nitroimidazole (metronidazole or tinidatzole)	7	(2)
Amoxicillin	5	(1)
Tetracycline	1	(0)
Other / not known	25	(5)
Two or more antimicrobials (without doxycycline)	15	(3)
Treatment for malaria (missing data = 0) ^e^	4	(1)
Gastrointestinal medication (missing data = 0)	127	(28)
Loperamide	115	(25)
Medicinal charcoal	7	(2)
Other (laxative, spasmolyte, co-phenotrope)	10	(2)
Two or more gastrointestinal medications	5	(1)
Analgesic (missing data = 0)	123	(27)
NSAID	67	(15)
Paracetamol	59	(13)
Other / not known	16	(3)
Two or more analgesics	18	(4)
Antiemetic (missing data = 0)	4	(1)
PPI or antacid (missing data = 4)	36	(8)
PPI	26	(6)
Antacid (salts/H2)	10	(2)
Probiotics and prebiotics (missing data = 3) ^f^	270	(59)
Starting before travel	11	(2)
Using only during travel	123	(27)
Starting before and using during travel	136	(30)

^a^ Total use of all travelers among those from whom information was available

^b^ Each of these changed the medication from doxycycline to mefloquine

^c^ Did not use as recommended (2 did not take at all, 1 stopped because of adverse effects, 4 used irregularly): atovaquone/proguanil (4), doxycycline (2), mefloquine (1); 10 had prescription but did not enter malaria area; 33 traveled to endemic countries yet visited only low prevalence areas

^d^ 52 (11 % of all) used antimicrobials for TD

^e^ All in Africa, three of four were tested; two negative, one positive

^f^ If only product mentioned, presumed to be used while traveling

### Travel information and behavior

The 460 travelers visited 77 countries altogether (UN definition); India (64), Thailand (60), Gambia (50), Tanzania (48), and Kenya (40) ranking as their most popular destinations. Overall 662 country visits were made (average 1.45 per person; range 1–8). The 30 most favored countries accounted for 560 journeys (85 %); only three destinations were in developed regions (UN definition [33]): USA (8), Spain (7), and the Netherlands (5).

Uncooked meat/fish (Table 4) was eaten more frequently by young adults (18–35 years, 17 %) and the middle-aged (36–55 years, 13 %) than children (0–17 years, 6 %) and older travelers (56– years, 6 %) (*p* = 0.016). Young adults proved more likely than the others to have freshwater contact, neglect hand washing, and not to shun salads or eating without utensils. Men consumed more alcohol than women (*p* < 0.001).

**Table 4:** Traveler profiles presented by demographics (destination, sex, age, duration of travel, purpose of travel) with travel behavior in bivariable analysis for 460 travelers. Results are given in percentages with the largest group shown in bold

	Number (%)	Destination %				Sex %			Age group (years) %					Duration of travel (days) %				Purpose of travel %			
		Africa	Asia	other	*p*-value	male	female	*p*-value	0–17	18–35	36–55	56–	*p*-value	1–7	8–29	30–160	*p*-value	vacation	business	other	*p*-value
Age group (missing data = 0)					<0.001*			0.833									<0.001*				0.056
0–17	35 (8)	8	7	11		8	7							30	7	3		9	0	3	
18–35	196 (43)	**32**	**55**	**41**		**43**	**42**							22	**36**	**72**		**42**	37	**61**	
36–55	120 (26)	30	23	23		27	25							**33**	29	13		26	**39**	16	
56–	109 (24)	31	15	25		21	25							15	28	12		24	24	21	
Duration of travel, days (missing data = 0)					<0.001*			0.473					<0.001*								<0.001^d^*
1–7	27 (6)	2	6	20		7	5		23	3	8	4						6	13	0	
8–29	334 (73)	**81**	**63**	**73**		**72**	**73**		**69**	**61**	**82**	**85**						**79**	**53**	29	
30–160	99 (22)	17	31	7		20	22		9	36	11	11						15	34	**71**	
Purpose of travel (missing data = 1) ^a^					0.034^d^*			0.002*					0.056				<0.001^d^*				
vacation	383 (84)	**78**	**88**	**91**		**86**	**82**		**97**	**81**	**83**	**84**		**81**	**91**	**60**					
business	37 (8)	12	5	4		11	7		0	7	13	8		19	6	13					
other/multiple	35 (8)	10	7	5		3	11		3	12	5	7		0	3	27					
Accommodation (missing data = 11) ^b^					<0.001*			0.008*					<0.001*				<0.001*				<0.001*
hotel	188 (42)	**39**	40	**61**		**46**	**40**		34	31	**50**	**55**		**85**	**46**	15		**44**	**45**	18	
home of a local	71 (16)	23	9	14		9	20		26	16	11	17		15	12	29		12	16	**50**	
guest house	190 (42)	**39**	**52**	25		45	41		**40**	**53**	39	28		0	42	**56**		**44**	39	32	
Location (missing data = 22) ^b^					<0.001*			0.083					0.088				<0.001*				0.494
city	143 (33)	24	34	**57**		28	36		44	27	36	38		**88**	31	23		33	38	25	
countryside/jungle	295 (67)	**76**	**66**	43		**72**	**64**		**56**	**73**	**66**	**62**		12	**69**	**77**		**67**	**62**	**75**	
Used other than bottled water (missing data = 3) ^b^	25 (5)	8	3	5	0.144	6	5	0.499	3	4	8	6	0.373	0	5	7	0.336	5	11	5	0.379^d^
Ate uncooked meat/fish (missing data = 4)	58 (13)	9	16	18	0.045*	11	14	0.446	6	17	13	6	0.016*	4	12	18	0.080	13	13	11	0.961^d^
Ate salads (missing data = 26)	341 (79)	77	78	85	0.427	79	78	0.904	59	83	81	74	0.009*	69	78	83	0.288	80	70	78	0.404
Diet (missing data = 159)					0.087			0.018*					0.010*				0.288				1.000^d^
omnivore	274 (91)	**92**	**88**	100		**96**	**88**		**100**	**85**	**92**	**97**		**100**	**92**	**88**		**91**	**91**	**93**	
vegetarian	27 (9)	8	12	0		4	12		0	15	8	3		0	8	13		9	9	7	
Used milk as part of diet (missing data = 161)	293 (98)	99	97	96	0.226^d^	99	97	0.426^d^	96	98	99	97	0.599^d^	100	99	96	0.347^d^	98	100	100	1.000^d^
Site of meals (missing data = 12) ^b^					<0.001*			0.099					<0.001*				0.660				0.042*
restaurant more than 50 %	372 (83)	**70**	**96**	**87**		**87**	**81**		**53**	**86**	**86**	**83**		**89**	**83**	**81**		**84**	**83**	**68**	
own household and sometimes elsewhere	76 (17)	30	4	13		13	19		47	14	14	17		11	17	19		16	17	32	
Alcohol consumption (missing data = 67) ^c^					0.112			<0.001*					0.001*				0.117				0.025*
0–2 units per day	281 (61)	**72**	**74**	**59**		**61**	**78**		**100**	**70**	**64**	**71**		**65**	**69**	**80**		**70**	**64**	**91**	
3– units per day	112 (24)	28	26	41		39	22		0	30	36	29		35	31	20		30	36	9	
Did not always use utensils (missing data = 23)	126 (29)	28	34	18	0.070	28	29	0.877	39	40	24	10	<0.001*	17	22	55	<0.001*	26	39	49	0.004*
Did not wash hands always/often (missing data = 13)	61 (14)	10	16	18	0.147	17	12	0.138	9	21	9	7	0.001*	11	13	17	0.573	14	14	11	0.840
No WC as a toilet (missing data = 7)	74 (16)	22	13	5	0.004*	17	16	0.747	16	19	18	10	0.235	0	16	21	0.037*	16	14	24	0.411
Had fresh water contact (missing data = 160)	109 (36)	27	47	28	0.001*	38	35	0.584	26	50	33	14	<0.001*	6	31	59	<0.001*	37	24	46	0.243
Walking barefoot often/sometimes (missing data = 7)	322 (71)	64	80	69	0.002*	74	69	0.326	88	79	68	55	<0.001*	65	70	77	0.359	74	59	55	0.015*
Unprotected sex with local (missing data = 27)	8 (2)	4	1	0	0.067^d^	2	2	1.000^d^	0	1	4	1	0.239^d^	0	2	3	0.632^d^	2	0	3	0.587^d^
Other close contact with local (kissing / sharing meals) (missing data = 26)	82 (19)	23	17	9	0.055	17	20	0.336	18	25	19	8	0.007*	8	15	36	<0.001*	17	24	33	0.039*
Had insect stings (missing data = 22)	335 (76)	75	81	67	0.058	75	77	0.657	59	88	75	62	<0.001*	19	77	93	<0.001*	76	65	91	0.032*

^a^ 14 (3 %) reported two or more purposes of travel

^b^ Categorized according to lowest standard

^c^ Missing data for children under 16 years was replaced with 0 alcohol servings per day

^d^ Fisher's exact test used

* *P*-value less than 0.05

### Traveler profiles

#### Traveler profiles by age

The median duration of travel was 23 days for the young adults and 15 days for others (age group vs travel duration group *p* < 0.001). Young adults and children stayed most often in guest houses, while the others preferred hotel accommodation (Table 4).

#### Traveler profiles by destinations

To obtain data on typical travelers to the two most preferred geographic regions, Africa and Asia, the destinations were categorized into three subgroups: Africa (*n* = 212), Asia (*n* = 192), and others (*n* = 56). Their characteristics are presented in Table 4. In bivariable analysis, distribution of age and duration of journey differed between those heading for Africa and those favoring Asia. The median age was higher for those visiting Africa, and the journey was shorter. Likewise, compared to Asia, visitors to Africa had less frequent freshwater contact and ate uncooked meat/fish less often.

### Illness

Factors with *p* < 0.1 in the bivariable analyses (Table 5) were included in the multivariable analyses (Table 6). Destination associated strongly with health problems. Females were more predisposed to falling ill. Overall, the risk was greatest at 31.5 years of age. The longer the travel, the greater the risk of contracting an illness – it increased each day by 2.5 %. Eating raw meat/fish was associated with healthier travelers.

**Table 5:** Risk factors of contracting illness while traveling / on arrival among 460 travelers. Values are given for proportions of ill travelers with a given risk factor, odds ratios with 95 % confidence intervals, and *p*-values in a bivariable analysis

	Contracted illness ^a^ %	Odds ratio (95 % confidence interval)		*p*-value
Total (missing data = 1)	79			
Sex (missing data = 0)				
Male	75	1.0		
Female	81	1.5	(1.0–2.4)	0.079*
Age group (missing data = 0)				<0.001*
0–17	66	1.0		
18–35	89	4.3	(1.9–9.9)	0.001
36–55	73	1.4	(0.6–3.2)	0.380
56–	71	1.3	(0.6–2.8)	0.582
Geographic region (missing data = 0)				<0.001*
Europe, Northern America	20	1.0		
Latin America and the Caribbean	66	7.7	(1.9–32.0)	0.005
Northern Africa, Western Asia	67	8.0	(1.4–45.8)	0.019
Southern Africa	62	6.4	(1.4–28.4)	0.015
Western Africa, Middle Africa	79	14.9	(3.8–58.5)	<0.001
Eastern Africa	84	21.6	(5.4–85.8)	<0.001
Eastern Asia, Central Asia	56	5.0	(0.8–31.0)	0.084
South-Eastern Asia	87	26.6	(6.7–106.1)	<0.001
Southern Asia	91	41.3	(9.1–188.5)	<0.001
Duration of travel, days (missing data = 0)				<0.001*
1–7	52	1.0		
8–29	77	3.0	(1.4–6.7)	0.006
30–160	94	14.4	(4.7–44.1)	<0.001
Purpose of travel (missing data = 1)				0.813
Vacation	78	1.0		
Business	82	1.2	(0.5–2.9)	0.636
Other/multiple ^b^	82	1.2	(0.5–2.9)	0.636
Accommodation (missing data = 11) ^c^				<0.001*
Hotel	68	1.0		
Home of a local	87	3.2	(1.5–6.8)	0.003
Guest house	87	3.1	(1.8–5.2)	<0.001
Location (missing data = 22) ^c^				
City	71	1.0		
Countryside/jungle	83	2.0	(1.3–3.3)	0.003*
Used other than bottled water (missing data = 3) ^c^	76	0.8	(0.3–2.2)	0.710
Ate uncooked meat/fish (missing data = 4)	66	0.4	(0.2–0.8)	0.006*
Ate salads (missing data = 26)	78	0.7	(0.4–1.2)	0.192
Diet (missing data = 159)				
Omnivore	82	1.0		
Vegetarian	89	1.8	(0.5–6.2)	0.353
Used milk as part of diet (missing data = 161)	83	1.0	(0.1–8.5)	1.000^e^
Site of meals (missing data = 12) ^c^				
Restaurant more than 50 % of meals	79	1.0		
Own household and sometimes elsewhere	79	1.0	(0.5–1.8)	0.996
Alcohol consumption (missing data = 67) ^d^				
0–2 units per day	81	1.0		
3– units per day	74	0.7	(0.4–1.1)	0.102
Did not always use utensils (missing data = 23)	85	1.7	(1.0–3.0)	0.058*
Did not wash hands always/often (missing data = 13)	72	0.6	(0.3–1.2)	0.138
No WC as a toilet (missing data = 7)	85	1.6	(0.8–3.2)	0.159
Had fresh water contact (missing data = 160)	84	1.7	(0.9–3.1)	0.109
Walking barefoot often/sometimes (missing data = 7)	79	1.0	(0.6–1.7)	0.990
Unprotected sex with local (missing data = 27)	100	1.0	(1.0–1.0)	0.213^e^
Other close contact with local (missing data = 26)	88	2.2	(1.1–4.4)	0.030*
Had insect stings (missing data = 22)	84	3.1	(1.9–5.1)	<0.001*

^a^ Symptoms started abroad or on return

^b^ 14 (3 %) reported two or more purposes of travel

^c^ Categorized according to lowest standard

^d^ Missing data for children under 16 years was replaced with 0 alcohol servings per day

^e^ Fisher's exact test used

* Factors with *P*-value <0.1 chosen to multivariable model

**Table 6:** Risk factors of contracting illness while traveling / on return in the final multivariable model after backward selection of factors by Akaike information criteria^a^. Variables with *p*-value less than 0.10 in bivariable analysis were chosen to multivariable model. Values are given for proportions with a given risk factor, adjusted odds ratios with 95 % confidence intervals, and *p*-values in multivariable analysis

	Proportion of those contracting	AOR (95 % CI) for contracting illness among	
	illness among travelers with	travelers with the given risk factor in multivariable	
	the given risk factor (%)	analysis with multiple imputations ^c^	*p*-value
Gender			
male	75	1.00	
female	81	1.73 (1.03–2.91)	0.040*
Age, years ^b^	*N/A*	1.06 (0.994–1.14)	0.076
Age, quadratic term ^b^	*N/A*	0.999 (0.998–1.00)	0.018*
Geographic region			*N/A*
Europe, Northern America	20	1.00	
Latin America and the Caribbean	66	7.90 (1.66–37.6)	0.009*
Northern Africa, Western Asia	67	11.9 (1.82–78.0)	0.010*
Southern Africa	62	9.72 (1.85–51.2)	0.007*
Western Africa, Middle Africa	79	15.5 (3.50–68.7)	<0.001*
Eastern Africa	84	18.5 (4.08–83.8)	<0.001*
Eastern Asia, Central Asia	56	6.41 (0.930–44.2)	0.059
South-Eastern Asia	87	21.2 (4.48–100)	<0.001*
Southern Asia	91	32.4 (6.24–168)	<0.001*
Duration of travel, days ^b^	*N/A*	1.025 (1.00–1.05)	0.048*
Accommodation			*N/A*
hotel	68	1.00	
home of a local	87	2.36 (0.922–6.06)	0.073
guest house	87	1.78 (0.940–3.36)	0.077
Eating uncooked meat/fish			
did not eat uncooked meat/fish	81	1.00	
ate uncooked meat/fish	66	0.303 (0.147–0.625)	0.001*

*Abbreviations*: *AOR* adjusted odds ratios, *CI* confidence interval, *N/A* not applicable

^a^Backward selection eliminated the following factors: location, other close contact with local, having insect stings, and use of utensils

^b^Analyzed as continuous variables. Age also on quadratic term, which seems to be better than age only in the model by AIC

^c^10 datasets used in imputation

**Fig. 2:**
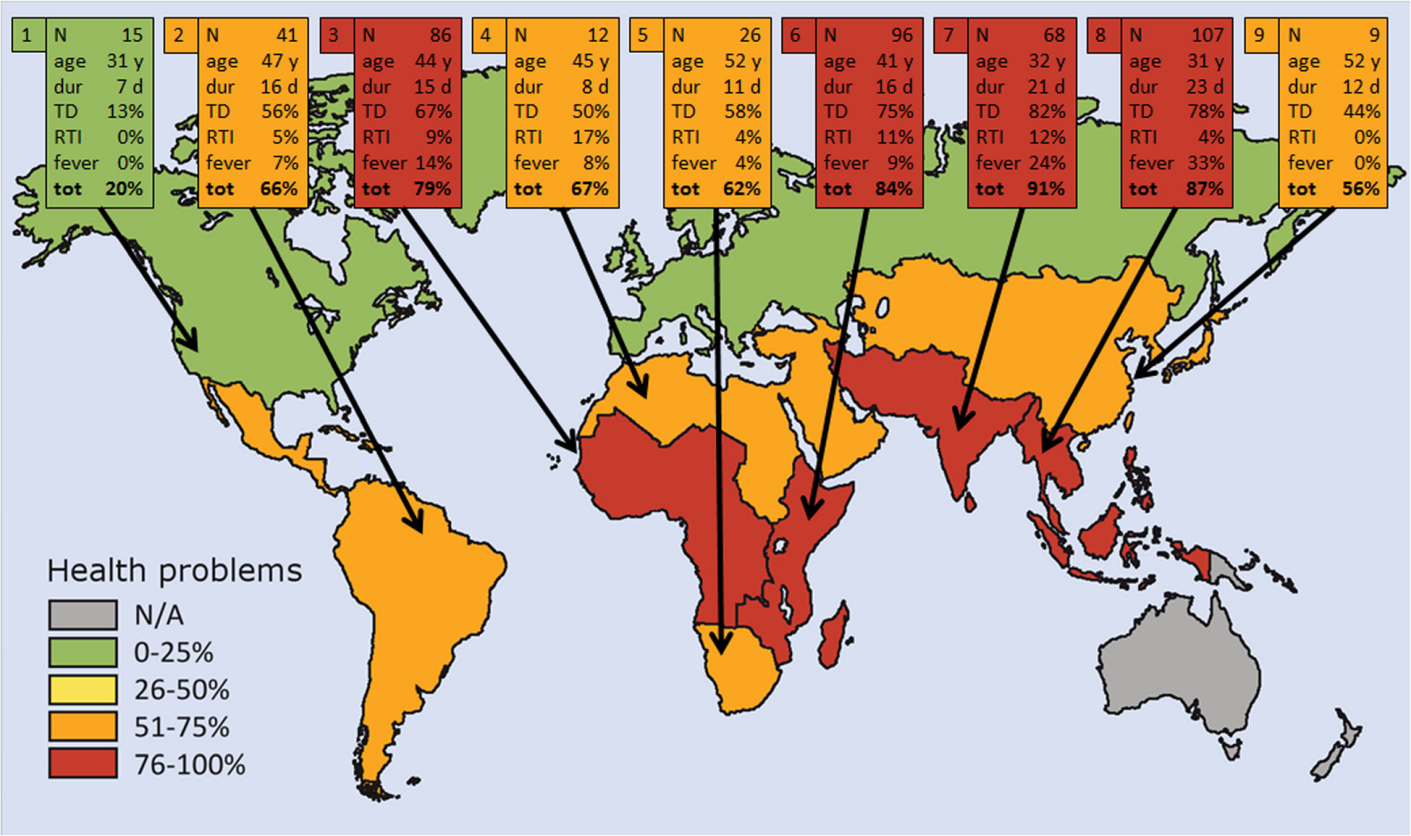
Demographics and illnesses of the 460 travelers. Each box presents data from one of the nine destinations by reporting the number of visitors, their median age in years, median duration of travel in days, and percentages of traveler with diarrhea (TD), respiratory tract infection (RTI), fever, or any symptom while traveling / on arrival. Map graphics devised by Helena Schmidt, HumanArt. N/A = not applicable.1 Europe and Northern America. 2 Latin America and the Caribbean. 3 Western Africa and Middle Africa. 4 Northern Africa and Western Asia. 5 Southern Africa. 6 Eastern Africa. 7 Southern Asia. 8 South-Eastern Asia. 9 Eastern Asia and Central Asia

Geographic regions with demographics and proportions of sick travelers are presented in Fig. 2. Illness rates by geographic regions were found to accord with respective risk ratings by International SOS (Additional file 2: Figure S2).

Health care was sought by 10 % of all subjects and by 13 % of those fallen ill during travel; 10 % and 13 % visited a physician and 0.7 % and 0.9 %, respectively, were hospitalized (Table 7). The most common reason for consulting health care professionals while abroad was TD – 4 % of all travelers visited a doctor, and 0.4 % were subsequently admitted to hospital because of this illness.

**Table 7:** Symptoms and contacts to health care providers. Data are provided for 459 travelers as numbers and percentages separately for the period during travel, on return, and one month after it

	During journey, N	Ongoing or started on arrival, N	Newly onset during follow-up, N
	N/459 (%^a^)	N/453 (%^a^)	N/233 (%^a^)
Any symptom	351 (76)	113 (25)	74 (32)
travelers' diarrhea	312 (68)	78 (17)	5 (2)
skin problem ^b^	78 (17)	2 (0)	9 (4)
fever >37,5 °C	74 (16)	6 (1)	20 (9)
vomiting	55 (12)	2 (0)	1 (0)
respiratory tract infection	26 (6)	17 (4)	9 (4)
urinary tract infection	17 (4)	1 (0)	4 (2)
ear infection	5 (1)	4 (1)	1 (0)
other gastrointestinal symptoms	19 (4)	2 (0)	4 (2)
other symptoms	16 (3)	6 (1)	40 (17)
Contacts to health care	47 (10)	1 (0)	50 (21)
Outpatient contact with health care ^c^	44 (10)	1 (0)	46 (20)
travelers’ diarrhea	20 (4)	0 (0)	16 (7)
skin problem ^d^	9 (2)	0 (0)	5 (2)
respiratory tract infection	8 (2)	0 (0)	5 (2)
ear infection	4 (1)	0 (0)	1 (0)
malaria / suspected malaria	3 (1)	0 (0)	0 (0)
musculosceletal problem	2 (0)	0 (0)	6 (3)
urinary tract infection	2 (0)	0 (0)	2 (1)
other reason	3 (1)	1 (0)	11 (5)
Inpatient (hospitalization)	3 (1)	0 (0)	4 (2)
travelers’ diarrhea	2 (0)	0 (0)	1 (0)
fever >37,5 °C	0 (0)	0 (0)	1 (0)
respiratory tract infection	0 (0)	0 (0)	1 (0)
suspected malaria	0 (0)	0 (0)	1 (0)
other reason	1 (0)	0 (0)	0 (0)

^a^ Proportion of positive among those from whom information was available

^b^ Dry skin / atopy / acne / insect stings excluded, sunburn / sun rash / infected skin due to insect stings included

^c^ Five travelers reported two or more causes of visit

^d^ Including travelers seeing a doctor because of any skin problem (*e.g*. insect stings, allergic reactions)

During follow-up, about one-third reported new-onset health problems (Table 7). Their most common single symptom was fever, followed by respiratory tract infection, skin problems, and TD.

## Discussion

### Introduction

Investigations addressing travelers’ health have generally been retrospective and/or conducted on those seeking medical care after their journeys, whereas prospective studies are scarce. Furthermore, research tends to focus on the effects that one or just a few specific factors have on travelers’ behavior and illness: destination [4, 26, 27], length of journey [4, 5, 26, 27], purpose of travel [27], gender [4, 5, 26, 27], age [4, 5, 26, 27], risk behavior [5], and particular diseases, such as TD [34–41]. We are not aware of any earlier investigations into all these factors with a single prospective cohort of travelers; only one [4] provides data on the same subjects during and after travel, and at follow-up.

### Morbidity during travel and on return

A central finding of our work is high morbidity rate. Despite efficient prophylactic measures taken before travel – exemplified by our vaccination data – the amount of health problems proved striking: as many as 76 % of our subjects reported illness while abroad, and 25 % still had ongoing symptoms or new complaints within two days after returning home. The overall proportion of our subjects with any symptoms while overseas (76 %) correspond to that reported for American (64 %; [4]), U.K. (64 %; [25]), and Israeli travelers (70 %; [5]), but proved higher than percentages presented for German (10-43 %; [26, 27]), Swiss (38 %; [24]), and Swedish travelers (49 %; [23]). As all these investigations are based on questionnaires, the format of the various questions concerning symptoms may account for the differences.

As regards morbidity in different geographic regions, the percentage of illness proved, as expected, significantly smaller for travelers to advanced countries (20 %) than those visiting developing regions (81 %); the rates were highest for visitors to Southern Asia, South-Eastern Asia, and Eastern Africa. TD accounted for the majority of health problems while abroad / after return (69 %). This result accords with several previous studies [4–6, 8, 10, 21–24, 26, 27, 42–44].

### Morbidity at follow-up

While the symptoms of illness mostly set in abroad, the figures proved unexpectedly high also during the follow-up: new health problems were reported by 32 % of our subjects. This percentage slightly exceeds the result of the only previous study exploring illness on return and at follow-up; the morbidity rate reported by Hill is 26 % [4]. The duration of follow-up may account for the small difference. In our study it was somewhat shorter (three weeks vs two months). But since the subjects were not asked to give exact dates for their symptoms, the onset could afterwards not be limited to 14 days in cases of TD, respiratory illness, and skin disorders, like in Hill’s report.

At follow-up, the four leading symptoms were fever, skin problems, respiratory illness, and TD, a finding according with Hill’s results [4]. In our study, fever was the most frequent single symptom – often associated with TD. Diarrhea remained the most common cause for seeking health care.

### Profiles

The data revealed regional differences which enabled profiling our subjects: travelers to Africa tended to be older and more cautious than those visiting Asia, whereas visitors to Asia were typically younger and favored longer trips. Likewise, the data show differences between travelers by age: the young traveled for longest and stayed in guest houses more frequently than the others, were the most likely to eat uncooked meat/fish and salads, not to use utensils, to follow a vegetarian diet, to neglect hand washing, and not to avoid freshwater contact. Not surprisingly, the longer the stay overseas, the lower the degree of travelers’ compliance with hygiene instructions. Such traveler profiles can be used as a tool to target advice at various groups according to their special characteristics.

### Risk factors

Destination, gender, age, and duration of travel were shown by multivariable analysis to be factors predisposing to illness. Southern Asia proved the riskiest resort, as also reported in earlier studies [4, 26]. Female travelers proved to be at greater risk of acquiring symptoms (OR 1.7), a finding according with the results published by Hill [4]. In the present data, each day increased the risk of contracting illness by 2.5 %; the respective figure obtained by Hill was 3.1–3.7 % [4]. Young age has often been reported to be associated with illness [4, 5, 8, 23, 26, 27]; in our cohort the risk was highest at 31.5 years. Eating raw meat or fish proved to be a protective factor. The reasons for this are not obvious and can only be speculated on.

### Limitations of the study

The present investigation has some limitations which deserve to be discussed. Firstly, the results are not representative of all travelers, but of volunteers with pre-travel appointments at a travel clinic. Thus visitors to Africa and other developing regions were overrepresented. Secondly, due to small sample size, statistical analyses of some subgroups were poorly powered. The third limitation concerns the follow-up: the reliability of the conclusions suffers from the fact that only 51 % of our volunteers completed the questionnaire, and many delivered it later than requested. Importantly, however, even if it could be assumed that none of the remaining 49 % had been ill, the number of newly onset symptoms during follow-up remains substantial. The data would have benefited from a comparison to non-traveling controls. Our data collection can also be regarded as a limitation, for questionnaire-based studies may distort the results in various ways. Due to the common use of questionnaires, this tends to be characteristic of research into travelers’ health problems [3, 4, 6, 22–27, 42, 44–48]; a few previous reports include data collected by telephone surveys [2, 4, 5, 8, 27]. In our study, travel diaries may have improved the accuracy of data to some degree. The exact number of clients declining to participate was not recorded. However, based on the number of clients seen by the recruiting doctor and number of those recruited, we estimate that at the maximum 10 % of the potentially eligible clients declined.

### Our data in relation to previous estimates of travelers’ health problems

We recently reported incidences of illness and injury among more than 50 000 Finnish travelers visiting various regions [21] by relating cases recorded by an assistance organization to numbers of travelers to each region. The results were considered to cover the most severe cases. The present data complete that picture by showing even the mildest symptoms contracted. This puts the two studies into perspective. Since 90 % of our travelers did not contact health care, the proportion covered by our previous report may not amount to more than 10 % of the illness altogether – slightly less than estimated in that study [21]. The strengths of the present research include the prospective study design and the fact that we combined data of three time points (pre-travel, travel/return, and follow-up) for a single cohort. Interestingly, the rate of health problems per region presented in our study accords with risk estimates presented by International SOS (Additional file 2: Figure S2).

### Aspects related to malaria prophylaxis and treatment

Advice on malaria prophylaxis is a cornerstone of pre-travel appointments. In our data, 289 of the 296 travelers (98 %) taking malaria prophylaxis reported compliance. Interestingly, however, four of them, all diagnosed in Africa, were also treated for malaria while abroad. One case was microbiologically verified, two reported a negative malaria test, and one had taken medication without laboratory diagnostics. Our earlier data collected by the assistance organization shows malaria to be rare in travelers (8/50000 cases; Siikamäki, personal communication). Indeed, the diagnoses of our cases may not be correct, as presumptive treatment is often given in Africa [49], and malaria diagnostics may not always be accurate [50].

## Conclusions

This study with most of its subjects visiting (sub)tropical regions shows that, despite efficient preventive measures like vaccinations, malaria prophylaxis, and travel advice, the majority fall ill during or after travel. TD is the most common disease while abroad, followed by skin problems and fever. After travel, the most frequent complaints are fever, respiratory tract infections, and skin problems. Symptoms generally remain mild, not requiring medical care. The proportion of newly onset illness among returning travelers is considerable: one-third get health problems after their journeys. Advice regarding this should be given already at pre-travel appointments.

## Abbreviations

TD, Travelers’ diarrhea

## Additional files

Additional file 1: Figure S1. HealthMap 2010 by International SOS. Medical risk ratings are based on the standard of local medical and dental care, access to prescription drugs, the possible prevalence of serious infectious diseases, and known cultural, linguistic and administrative barriers. Map printed with the written permission of International SOS. This map has been developed for illustrative purposes only. It is a global illustration of medical risk for travellers. For detailed information, please refer to the country guides at internationalsos.com © International SOS, 2010. All rights reserved. Unauthorized copy or distribution prohibited. (PDF 3143 kb) Additional file 2: Figure S2. HealthMap 2010 by International SOS presented together with demographics and illnesses of our 460 travelers. Each box presents data from one of the nine destinations by reporting the number of visitors, their median age in years, median duration of travel in days, and percentages of traveler with diarrhea (TD), respiratory tract infection (RTI), fever, or any symptom while traveling / on arrival. Map printed and modified with the written permission of International SOS. This map has been developed for illustrative purposes only. It is a global illustration of medical risk for travellers. For detailed information, please refer to the country guides at internationalsos.com © International SOS, 2010. All rights reserved. Unauthorized copy or distribution prohibited. 1 Europe and Northern America. 2 Latin America and the Caribbean. 3 Western Africa and Middle Africa. 4 Northern Africa and Western Asia. 5 Southern Africa. 6 Eastern Africa. 7 Southern Asia. 8 South-Eastern Asia. 9 Eastern Asia and Central Asia. (TIF 604 kb)
