# Supplementary material for: Travelers’ health problems and behavior: prospective study with post-travel follow-up
Source: BMC Infect Dis. 2016 Jul 13;16:328. doi: 10.1186/s12879-016-1682-0 (PMC4944265; doi:10.1186/s12879-016-1682-0)
Supplement: Additional file 1: Figure S1. — HealthMap 2010 by International SOS. Medical risk ratings are based on the standard of local medical and dental care, access to prescription drugs, the possible prevalence of serious infectious diseases, and known cultural, linguistic and administrative barriers. Map printed with the written permission of International SOS. This map has been developed for illustrative purposes only. It is a global illustration of medical risk for travellers. For detailed information, please refer to the country guides at internationalsos.com © International SOS, 2010. All rights reserved. Unauthorized copy or distribution prohibited. (PDF 3143 kb) [file 12879_2016_1682_MOESM1_ESM.pdf]

HEALTHMAP

2010 Understanding Global Health

International SOS™

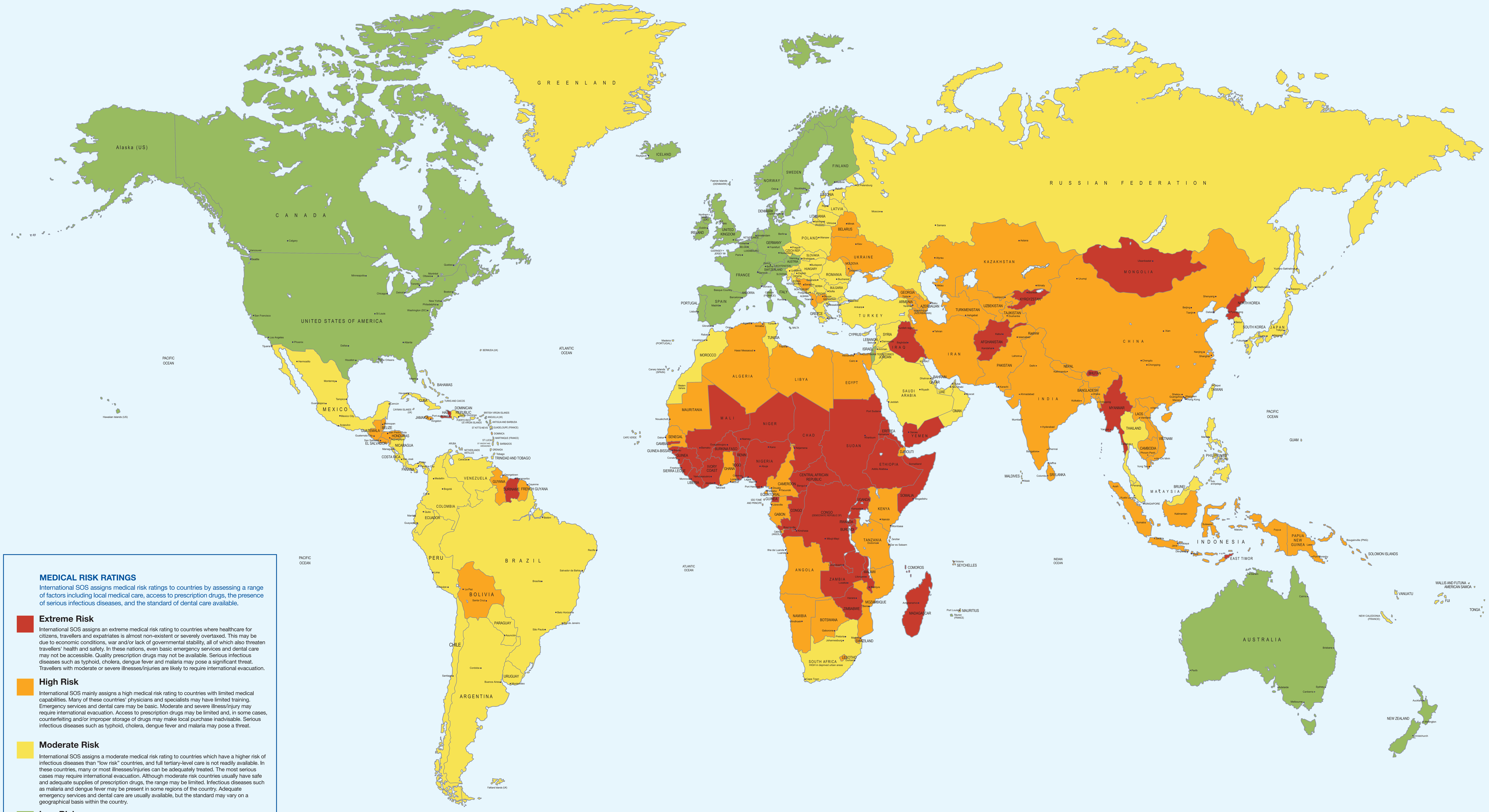

MEDICAL RISK RATINGS

International SOS assigns medical risk ratings to countries by assessing a range of factors including local medical care, access to prescription drugs, the presence of serious infectious diseases, and the standard of dental care available.

Extreme Risk

International SOS assigns an extreme medical risk rating to countries where healthcare for citizens, travellers and expatriates is almost non-existent or severely overtaxed. This may be due to economic conditions, war and/or lack of governmental stability, all of which also threaten travellers' health and safety. In these nations, even basic emergency services and dental care may not be accessible. Quality prescription drugs may not be available. Serious infectious diseases such as typhoid, cholera, dengue fever and malaria may pose a significant threat. Travellers with moderate or severe illnesses/injuries are likely to require international evacuation.

High Risk

International SOS mainly assigns a high medical risk rating to countries with limited medical capabilities. Many of these countries' physicians and specialists may have limited training. Emergency services and dental care may be basic. Moderate and severe illness/injury may require international evacuation. Access to prescription drugs may be limited and, in some cases, counterfeiting and/or improper storage of drugs may make local purchase inadvisable. Serious infectious diseases such as typhoid, cholera, dengue fever and malaria may pose a threat.

Moderate Risk

International SOS assigns a moderate medical risk rating to countries which have a higher risk of infectious diseases than “low risk” countries, and full tertiary-level care is not readily available. In these countries, many or most illnesses/injuries can be adequately treated. The most serious cases may require international evacuation. Although moderate risk countries usually have safe and adequate supplies of prescription drugs, the range may be limited. Infectious diseases such as malaria and dengue fever may be present in some regions of the country. Adequate emergency services and dental care are usually available, but the standard may vary on a geographical basis within the country.

Low Risk

International SOS assigns a low medical risk rating to nations that are capable of offering the best medical care in the world. In these countries, all tertiary-level and specialist and sub-specialist care is available. Physicians have been trained to “international” standards and medical boards control licensure. Most prescription drugs, or acceptable alternative brands, are available. Travellers in low risk countries usually face little risk of contracting serious infectious diseases, especially in urban areas. High quality emergency and dental services are available. It is unlikely that a visitor would require an international evacuation, as appropriate medical care would be available in-country for almost any medical problem.

This map has been developed for illustrative purposes only. It is a global illustration of medical risk for travellers. For detailed information, please refer to the country guides at internationalsos.com

© International SOS, 2010. All rights reserved. Unauthorized copy or distribution prohibited.

CR Risk Medicaux_Map 2010_1000x705_100PC_v7.indd 2
23/06/10 16:13
