# Supplementary figures and images for: Travelers’ health problems and behavior: prospective study with post-travel follow-up
Source: BMC Infect Dis. 2016 Jul 13;16:328. doi: 10.1186/s12879-016-1682-0 (PMC4944265; doi:10.1186/s12879-016-1682-0)

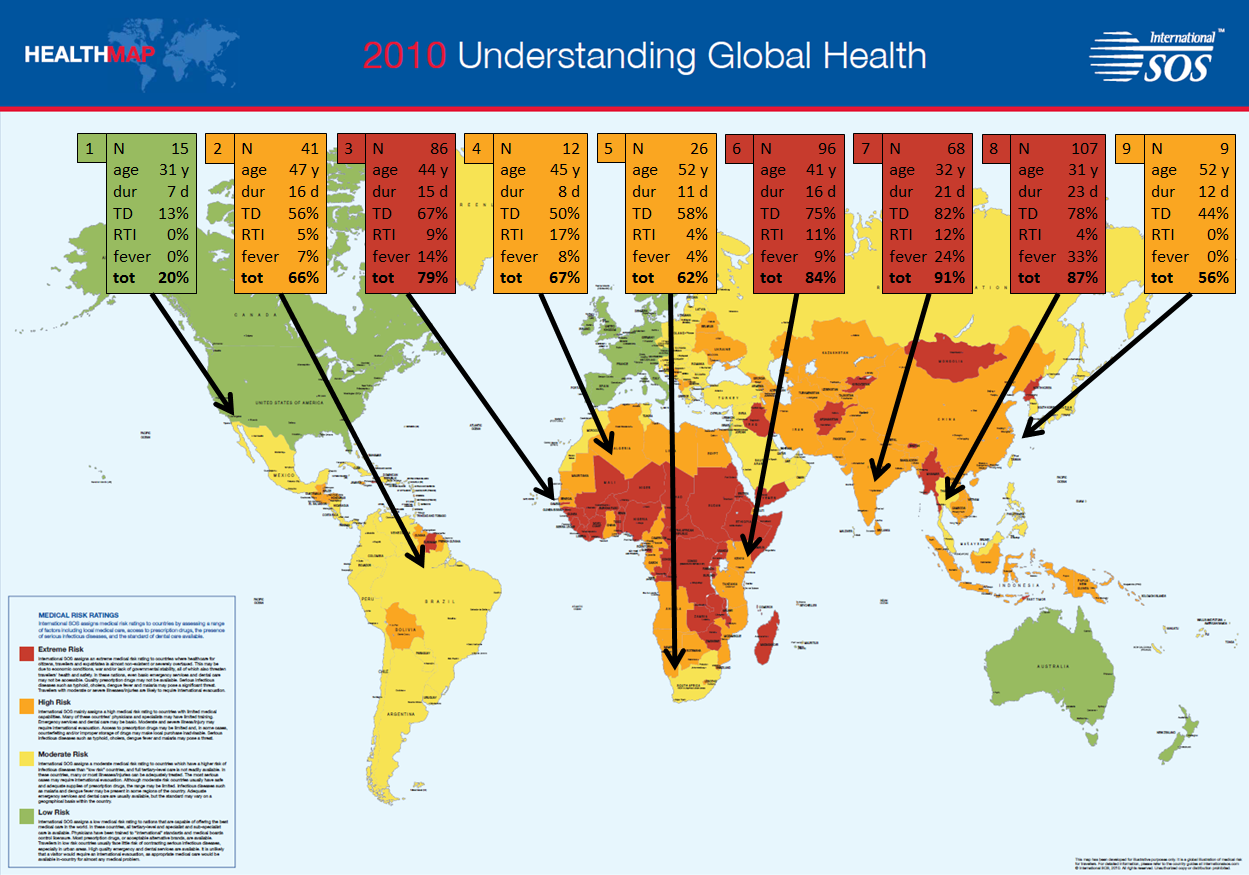

Supplement: Additional file 2: Figure S2. — HealthMap 2010 by International SOS presented together with demographics and illnesses of our 460 travelers. Each box presents data from one of the nine destinations by reporting the number of visitors, their median age in years, median duration of travel in days, and percentages of traveler with diarrhea (TD), respiratory tract infection (RTI), fever, or any symptom while traveling / on arrival. Map printed and modified with the written permission of International SOS. This map has been developed for illustrative purposes only. It is a global illustration of medical risk for travellers. For detailed information, please refer to the country guides at internationalsos.com © International SOS, 2010. All rights reserved. Unauthorized copy or distribution prohibited. 1 Europe and Northern America. 2 Latin America and the Caribbean. 3 Western Africa and Middle Africa. 4 Northern Africa and Western Asia. 5 Southern Africa. 6 Eastern Africa. 7 Southern Asia. 8 South-Eastern Asia. 9 Eastern Asia and Central Asia. (TIF 604 kb) [file 12879_2016_1682_MOESM2_ESM.tif]
